# Introduction of a leguminous shrub to a rubber plantation changed the soil carbon and nitrogen fractions and ameliorated soil environments

**DOI:** 10.1038/s41598-018-35762-0

**Published:** 2018-11-23

**Authors:** Chang-An Liu, Yu Nie, Yan-Ming Zhang, Jian-Wei Tang, Kadambot H. M. Siddique

**Affiliations:** 1 CAS Key Laboratory of Tropical Plant Resources and Sustainable Use, Xishuangbanna Tropical Botanical Garden, Chinese Academy of Sciences, Menglun town, Mengla county, Yunnan Province, 666303 China; 2 University of Chinese Academy of Sciences, Beijing, 100049 China; 3 Yunnan Institute for Food and Drug Control, Kunming, Yunnan Province, 650011 China; 4 The UWA Institute of Agriculture, The University of Western Australia, Crawley, WA 6009 Australia

## Abstract

The conversion of monoculture rubber (*Hevea brasiliensis*) plantations into rubber-based agroforestry systems has become a common trend in forestry management in the past few decades. Rubber–*Flemingia macrophylla* (a leguminous shrub) systems are popular in southwestern China’s Xishuangbanna region. The biogeochemical cycles of soil carbon and nitrogen in forests are mainly affected by their fractions. This study investigated the effect of introducing *Flemingia macrophylla* to rubber plantations of different ages on soil carbon and nitrogen fractions. The experimental treatments included R1 (young rubber plantation), RF1 (young rubber–*Flemingia macrophylla* system), R2 (mature rubber plantation) and RF2 (mature rubber–*Flemingia macrophylla* system). The results showed that the introduction of *Flemingia macrophylla* to rubber plantations of different ages significantly changed soil carbon and nitrogen fractions, improved soil labile organic carbon and nitrogen contents, and ameliorated soil environments. The average soil microbial biomass organic carbon, nitrogen and nitrate-nitrogen in the 0–10 cm soil layer during the experimental period was 38.9%, 55.5%, and 214.7% higher in RF1 than R1, respectively, and 22.1%, 22.2%, and 652.2% higher in RF2 than R2, respectively. Therefore, *Flemingia macrophylla* can be used as an alternative interplanted tree species within rubber plantations in similar environments of southeastern Asia.

## Introduction

Monoculture rubber (*Hevea brasiliensis*) plantations have rapidly expanded in the last few decades in southeastern Asia^1,2^. Approximately 90% of global natural rubber production is derived from plantations in this region (http://www.rubberstudy.com), accounted for an estimated 84% of the total global rubber plantation area in 2012^1,3^. Rapid growth in the Chinese economy has increased demand for natural rubber. In response to this demand, the natural tropical forests of southwestern China’s Xishuangbanna region were deforested and replaced with more than 470,000 ha of rubber plantations, which equates to more than 24% of the total land area of the region^4^. The expansion of these rubber plantations has led to water loss and soil erosion^5,6^, environmental degradation^3,7^, and threatened environmental biodiversity^8^. At present, rubber-based agroforestry systems are considered the best way to resolve the environmental problems associated with rubber monoculture. In recent years, the local government of Xishuangbanna proposed the development of environmentally friendly rubber plantations to reduce the water and soil losses and increase environmental biodiversity^9,10^.

Legume plants could greatly enhance ecosystem services. Lucerne (*Medicago sativa*) and erect milkvetch (*Astragalus adsurgens*) have the potential to phytoextract rhenium from coal fly ash-amended alkaline soils^11^. Alfalfa (*Medicago sativa* L.) can improve its phosphorus acquisition by increasing specific root length and exuding gcarboxylates into the rhizosphere in phosphorus-deficient environments^12^. A combination of legumes grass species can enhance soil C and N storage, productivity, and diversity in semi-arid grasslands^13^. *Flemingia macrophylla* is used in traditional medicine for various therapeutic uses and is widely planted in the Xishuangbanna area of China. *Flemingia macrophylla* is a perennial leguminous leafy shrub with strong biological nitrogen fixation and high biomass^14^. As a result, rubber–*Flemingia macrophylla* intercropped systems have become popular in the Xishuangbanna area to improve soil carbon and nitrogen storage.

Precise and accurate estimations of carbon and nitrogen levels in forest soil are important for understanding biogeochemical cycles^15–17^. Some studies have indicated that soil organic carbon (SOC) has been depleted in rubber plantations^18,19^. Rubber plantations have 15% lower annual surface soil CO_2_ fluxes than natural forests, because they have lower soil respiration during the dry season^20^. The conversion of tropical rainforests into rubber plantations has increased N_2_O emissions, which may potentially enhance local climate warming trends^21^. Rubber plantations have lower mean CH_4_ uptake rates than secondary and tropical forests^22^. These transformations and biogeochemical cycles of soil carbon and nitrogen are mainly affected by their fractions^23–27^ that play essential roles in the turnover of nutrients in soil, including water-soluble organic carbon (WSOC), light fraction of organic C (LFOC), microbial biomass organic C (MBC), ammonium N (AN), nitrate N (NN), light fraction of organic N (LFON), and microbial biomass organic N (MBN). These fractions are often used to study the impacts of land management and ecological succession^26,28,29^. Rubber-based agroforestry systems have higher SOC and nitrogen levels and lower carbon and nitrogen losses than rubber plantations due to improved soil macroaggregates^30^. However, little is known about the effect of these systems on soil carbon and nitrogen fractions.

The objectives of this study were to examine: (1) soil carbon and nitrogen fractions, and (2) the relationships between soil carbon and nitrogen fractionsin rubber and rubber–*Flemingia macrophylla* plantations of different ages.

## Results

### Soil carbon fractions

The SOC content in the 0–10 or 10–30 cm soil layers did not differ between the plantation treatments (R1, R2, RF1, RF2) for the duration of the study (April 2014 to January 2017) (Table 1). However, SOC content in the 0–10 cm soil layers was significantly higher than the 10–30 cm soil layers in each plantation type. In the 0–10 cm soil layer, RF1 had consistently higher WSOC content than R1 from June 2015 to January 2017, which differed significantly in August 2016 and January 2017. The WSOC content between R2 and RF2 did not differ significantly for the duration of the experiment (Table 2). In the 10–30 cm soil layer, the introduction of *Flemingia macrophylla* to the rubber plantations had no significant effect on WSOC content.

**Table 1:** Soil organic carbon (SOC) (g kg^−1^) in rubber and rubber–*Flemingia macrophylla* plantations from April 2014 to January 2017 (mean ± SD, *n* = 3).

Soil depth (cm)	Treatments	Apr 2014	Jun 2015	Jan 2016	Aug 2016	Jan 2017
0–10 cm	R1	12.50 ± 0.47abA	12.45 ± 0.31aA	12.64 ± 0.79abA	12.50 ± 0.34aA	12.77 ± 0.46abA
	RF1	12.77 ± 2.53aA	13.04 ± 1.23aA	13.22 ± 1.42aA	13.59 ± 1.77aA	13.45 ± 1.65aA
	R2	13.79 ± 0.31aA	13.53 ± 0.60aA	14.40 ± 1.02aA	14.11 ± 1.35aA	14.20 ± 0.41aA
	RF2	13.16 ± 0.90 aA	12.74 ± 0.97 aA	13.04 ± 0.68abA	12.76 ± 1.36 aA	13.24 ± 1.88 aA
10–30 cm	R1	9.89 ± 0.71cA	9.65 ± 0.06bA	9.73 ± 0.94cA	9.73 ± 0.43bA	9.98 ± 0.63cA
	RF1	10.49 ± 0.31cA	10.61 ± 1.02bA	10.25 ± 2.43cA	9.84 ± 1.85bA	10.08 ± 0.69cA
	R2	10.22 ± 0.33cA	10.90 ± 0.18bA	11.00 ± 0.49bcA	10.24 ± 0.42bA	10.67 ± 0.64cA
	RF2	10.84 ± 1.03bcA	10.51 ± 0.49bA	10.20 ± 0.37cA	11.16 ± 1.34bA	10.92 ± 1.32bcA

R1: rubber plantations established in 2006; R2: rubber plantations established in 1994; RF1: *Flemingia macrophylla* introduced to R1 in 2010; RF2: *Flemingia macrophylla* introduced to R2 in 2010.Values within a column followed by the same letter (lower case) or within the same row (upper case) do not differ significantly at *P* ≤ 0.05.

**Table 2:** Soil water-soluble organic carbon (WSOC) (mg kg^−1^) in rubber and rubber–*Flemingia macrophylla* plantations from June 2015 to January 2017 (mean ± SD, *n* = 3).

Soil depth (cm)	Treatments	Jun 2015	Jan 2016	Aug 2016	Jan 2017
0–10 cm	R1	70.7 ± 3.7b	78.3 ± 1.0a	67.5 ± 2.2b	67.7 ± 6.7b
	RF1	72.3 ± 1.9b	86.0 ± 5.5a	84.9 ± 7.0a	80.5 ± 7.8a
	R2	84.5 ± 6.1a	86.6 ± 10.1a	75.1 ± 5.0ab	50.1 ± 4.4d
	RF2	82.5 ± 1.7a	80.5 ± 2.7a	76.9 ± 6.2ab	57.7 ± 8.0bcd
10–30 cm	R1	71.2 ± 5.5b	80.7 ± 8.4a	72.8 ± 8.0b	66.1 ± 1.5b
	RF1	70.7 ± 7.3b	82.5 ± 4.2a	75.6 ± 7.1ab	63.2 ± 8.7bc
	R2	88.8 ± 3.4a	82.3 ± 8.2a	75.3 ± 4.6ab	50.4 ± 6.0d
	RF2	85.9 ± 2.3a	87.8 ± 14.8a	70.6 ± 7.7b	55.0 ± 1.2 cd

R1: rubber plantations established in 2006; R2: rubber plantations established in 1994; RF1: *Flemingia macrophylla* introduced to R1 in 2010; RF2: The *Flemingia macrophylla* introduced to R2 in 2010. Values within a column followed by the same letter do not differ significantly at *P* ≤ 0.05.

**Figure 1:**
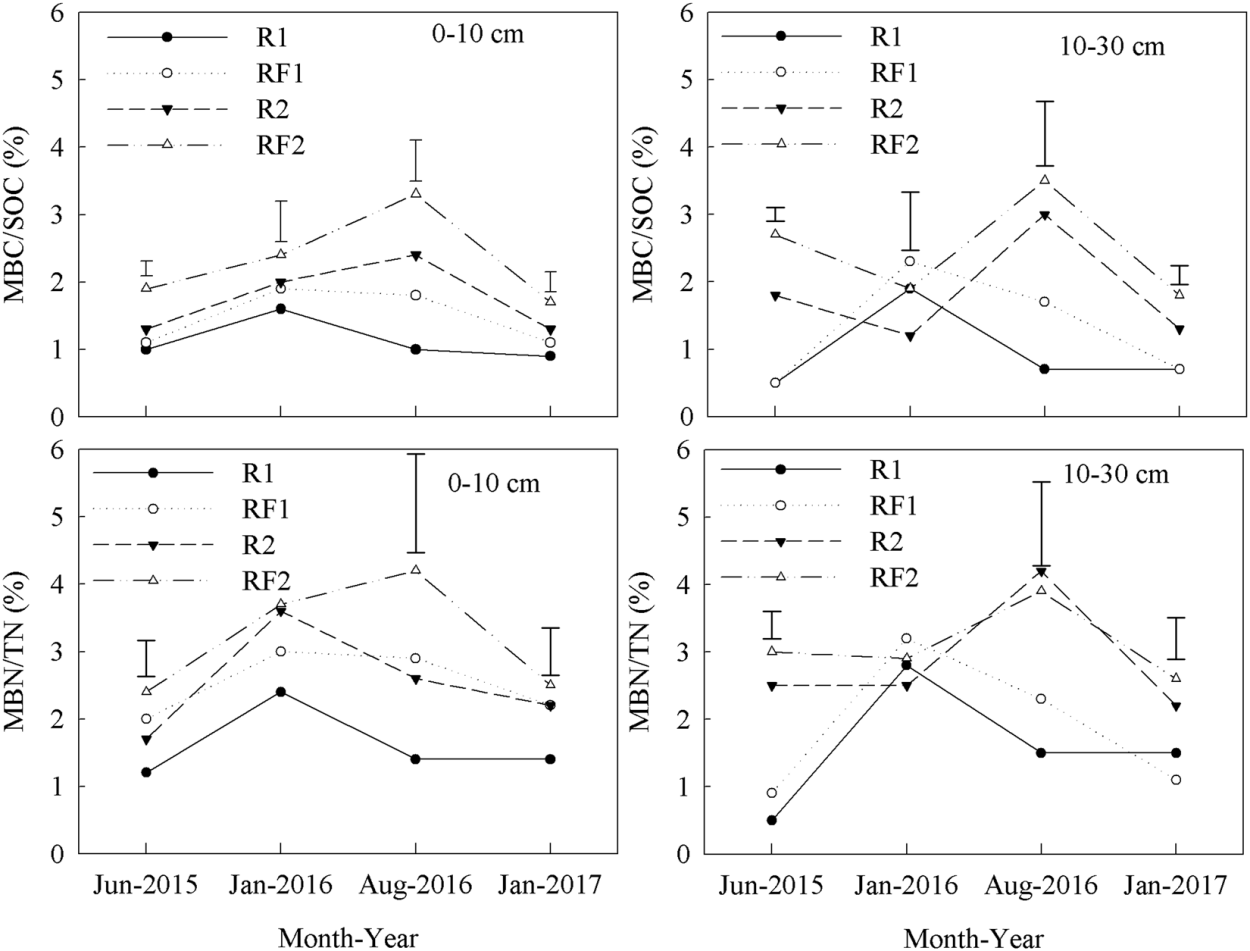
Ratio of soil microbial biomass carbon (MBC) to soil organic carbon (SOC) (MBC/SOC); and ratio of soil microbial biomass nitrogen (MBN) to soil total nitrogen (TN) (MBN/TN) in the rubber and rubber–*Flemingia macrophylla* plantations from June 2015 to January 2017. R1: rubber plantations established in 2006; R2: rubber plantations established in 1994; RF1: *Flemingia macrophylla* introduced to R1 in 2010; RF2: *Flemingia macrophylla* introduced to R2 in 2010. Vertical bars are LSD at *P* ≤ 0.05.

**Figure 2:**
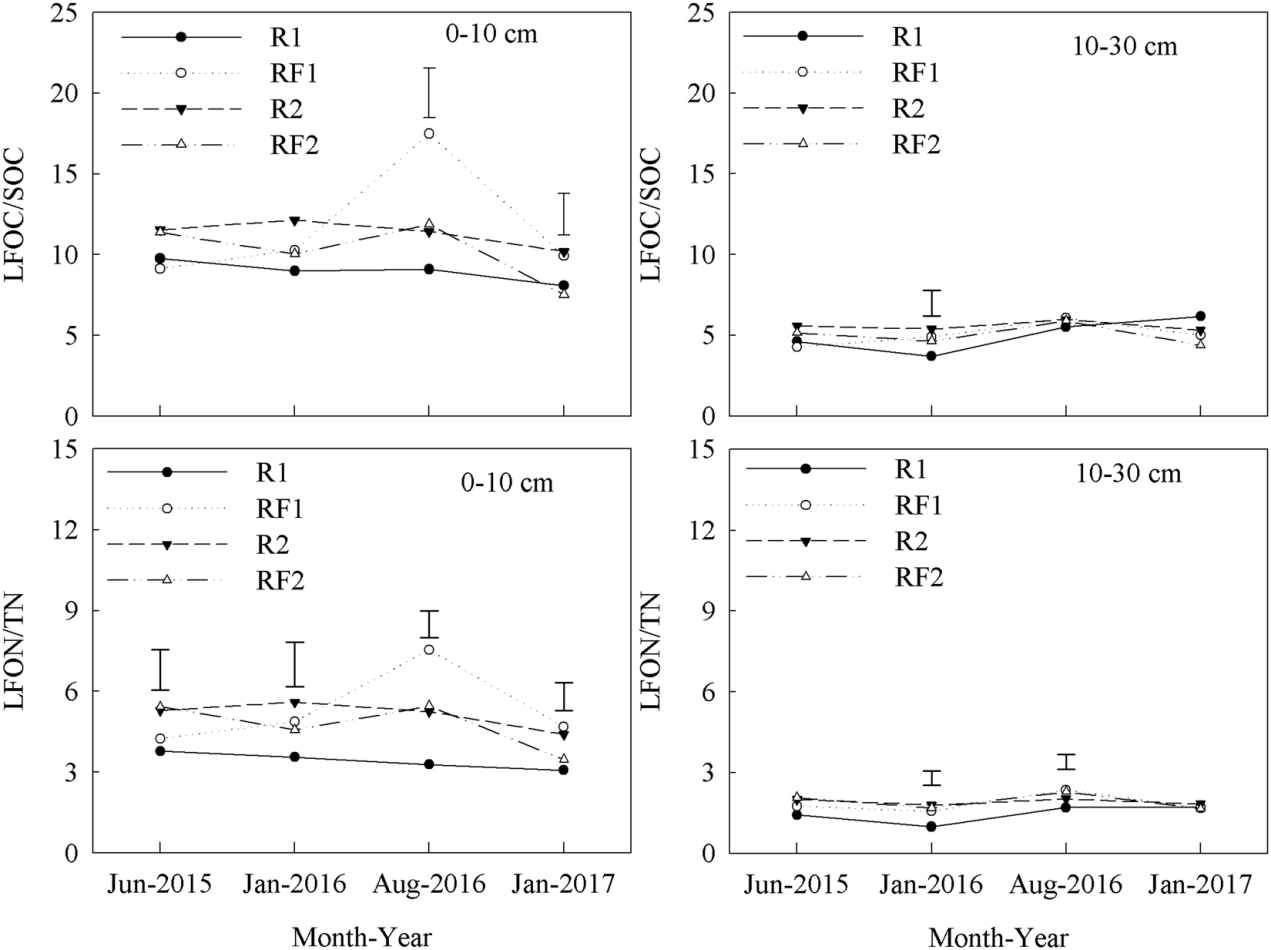
Ratio of soil light fraction carbon (LFOC) to soil organic carbon (SOC) (LFOC/SOC); and ratio of soil light fraction nitrogen (LFON) to soil total nitrogen (TN) (LFON/TN) in the rubber and rubber–*Flemingia macrophylla* plantations from June 2015 to January 2017. R1: rubber plantations established in 2006; R2: rubber plantations established in 1994; RF1: *Flemingia macrophylla* introduced to R1 in 2010; RF2: *Flemingia macrophylla* introduced to R2 in 2010. Vertical bars are LSD at *P* ≤ 0.05.

The 0–10 cm soil layer had significantly higher LFOC content than the 10–30 cm soil layer in each plantation type. In the 0–10 cm soil layer, RF1 had consistently higher LFOC content than R1 from June 2015 to January 2017, which differed significantly in August 2016 and January 2017. In the same layer, RF2 had consistently lower LFOC content than R2, with significant differences observed in January 2016 and 2017 (Table 3). In the 10–30 cm soil layer, the introduction of *Flemingia macrophylla* to the rubber plantations had no significant effect on LFOC content but increased MBC content and the ratios of MBC/SOC in the 0–10 and 10–30 cm soil layers (Table 4 and Fig. 1). In the 0–10 cm soil layer, RF1 had consistently higher ratios of LFOC/SOC than R1 from January 2016 to January 2017, but no significant differences were observed between the RF2 and R2 for the duration of the study (Fig. 2). In the 10–30 cm soil layer, the introduction of *Flemingia macrophylla* to the rubber plantations had no significant effect on the ratios of LFOC/SOC.

**Table 3:** Light fraction organic carbon (LFOC) (g kg^−1^) in rubber and rubber–*Flemingia macrophylla* plantations from June 2015 to January 2017 (mean ± SD, *n* = 3).

Soil depth (cm)	Treatments	Jun 2015	Jan 2016	Aug 2016	Jan 2017
0–10 cm	R1	1.21 ± 0.15b	1.13 ± 0.15b	1.14 ± 0.25c	1.03 ± 0.06b
	RF1	1.19 ± 0.10b	1.36 ± 0.21b	2.38 ± 0.23a	1.34 ± 0.11a
	R2	1.56 ± 0.13a	1.75 ± 0.30a	1.61 ± 0.26b	1.45 ± 0.14a
	RF2	1.45 ± 0.09a	1.31 ± 0.09b	1.51 ± 0.13b	1.00 ± 0.12b
10–30 cm	R1	0.45 ± 0.07c	0.36 ± 0.02c	0.54 ± 0.10d	0.61 ± 0.01c
	RF1	0.45 ± 0.07c	0.50 ± 0.07c	0.59 ± 0.06d	0.51 ± 0.16c
	R2	0.61 ± 0.09c	0.59 ± 0.12c	0.61 ± 0.11d	0.57 ± 0.18c
	RF2	0.54 ± 0.05c	0.47 ± 0.08c	0.60 ± 0.04d	0.48 ± 0.11c

R1: rubber plantations established in 2006; R2: rubber plantations established in 1994; RF1: *Flemingia macrophylla* introduced to R1 in 2010; RF2: *Flemingia macrophylla* introduced to R2 in 2010. Values within a column followed by the same letter do not differ significantly at *P* ≤ 0.05.

**Table 4:** Soil microbial biomass carbon (MBC) (mg kg^−1^) in rubber and rubber–*Flemingia macrophylla* plantations from June 2015 to January 2017 (mean ± SD, *n* = 3).

Soil depth (cm)	Treatments	Jun 2015	Jan 2016	Aug 2016	Jan 2017
0–10 cm	R1	122.0 ± 7.5e	203.9 ± 23.9de	126.5 ± 16.5de	115.6 ± 17.7c
	RF1	141.8 ± 15.0e	256.3 ± 24.3bc	245.0 ± 30.4c	145.9 ± 20.3c
	R2	174.3 ± 23.0d	281.3 ± 21.0ab	341.8 ± 25.7b	179.7 ± 17.6b
	RF2	236.1 ± 11.1b	314.0 ± 31.6a	420.7 ± 65.2a	222.4 ± 31.5a
10–30 cm	R1	47.4 ± 5.9f	182.2 ± 17.3e	72.1 ± 4.2e	68.2 ± 13.4d
	RF1	54.9 ± 11.8f	234.7 ± 18.1cd	164.4 ± 27.8d	70.3 ± 7.9d
	R2	199.7 ± 18.1c	136.9 ± 14.6f	310.6 ± 39.6bc	138.1 ± 17.9c
	RF2	280.9 ± 3.6a	192.8 ± 19.0e	354.5 ± 61.8ab	194.5 ± 7.2ab

R1: rubber plantations established in 2006; R2: rubber plantations established in 1994; RF1: *Flemingia macrophylla* introduced to R1 in 2010; RF2: *Flemingia macrophylla* introduced to R2 in 2010. Values within a column followed by the same letter do not differ significantly at *P* ≤ 0.05.

### Soil nitrogen fractions

**Figure 3:**
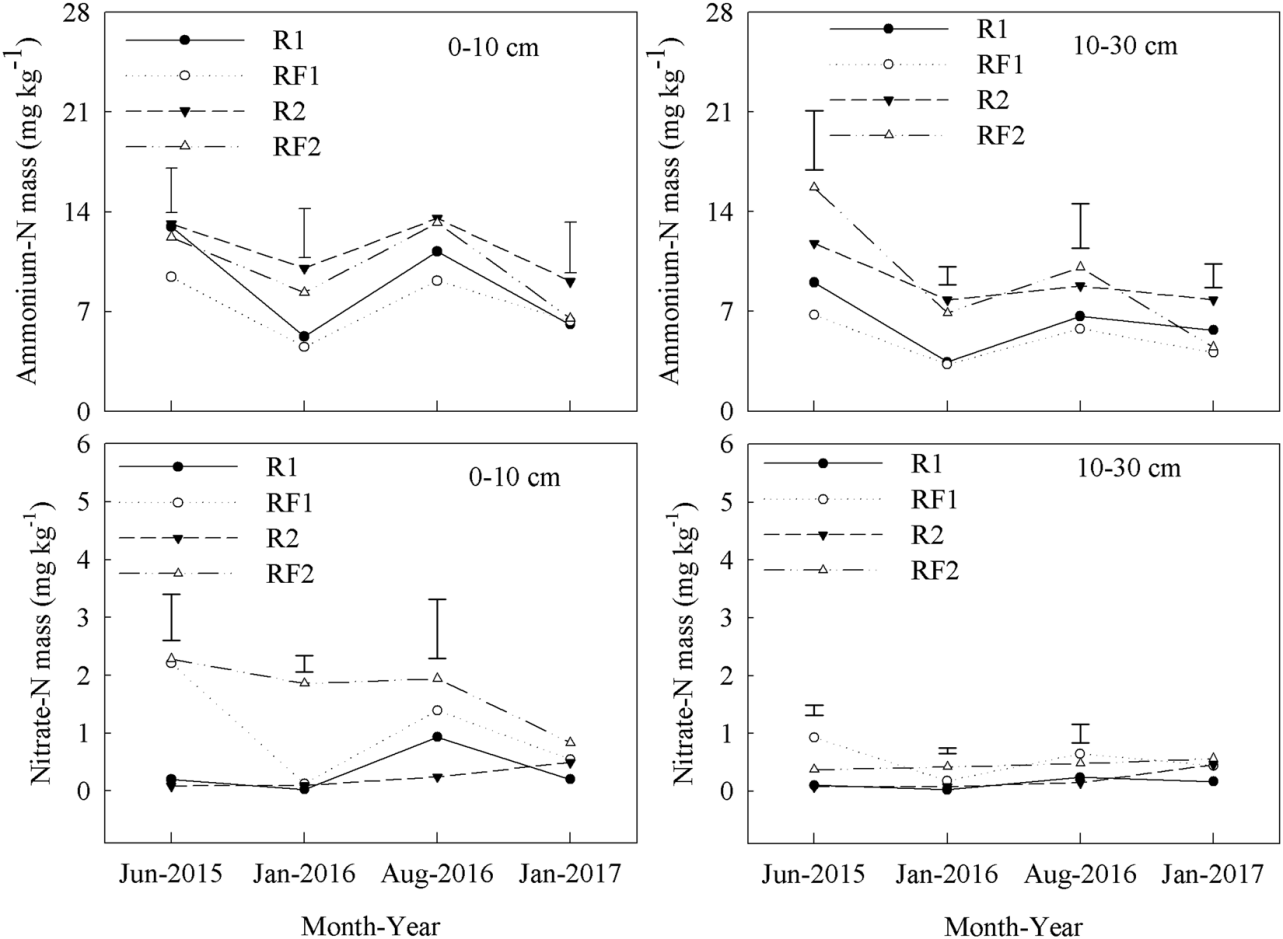
Soil ammonium N (AN) and nitrate N (NN) content levels in the rubber and rubber–*Flemingia macrophylla* plantations from June 2015 to January 2017. R1: rubber plantations established in 2006; R2: rubber plantations established in 1994; RF1: *Flemingia macrophylla* introduced to R1 in 2010; RF2: *Flemingia macrophylla* introduced to R2 in 2010. Vertical bars are LSD at *P* ≤ 0.05.

The TN content in the 0–10 or 10–30 cm soil layers did not differ between the plantation treatments (R1, R2, RF1, RF2) for the duration of the study (April 2014 to January 2017) (Table 5). However, the 0–10 cm soil layer had significantly higher TN contents than the 10–30 cm soil layer in each plantation type. The introduction of *Flemingia macrophylla* to the rubber plantations generally decreased AN content and increased NN content in the 0–10 and 10–30 cm soil layers (Fig. 3). In the 0–10 cm soil layer, RF1 had consistently higher LFON content than R1 from June 2015 to January 2017, with significant differences observed from January 2016 to January 2017. In the same layer, no significant differences in LFON content were observed between R2 and RF2, except for January 2017 when the LFON content was significantly higher in R2 than RF2 (Table 6). In the 10–30 cm soil layer, the introduction of *Flemingia macrophylla* to the rubber plantations had no significant effect on LFON content. The 0–10 cm soil layer had significantly higher LFON contents than the 10–30 cm soil layer in each plantation type.

**Table 5:** Soil total nitrogen (TN) (g kg^−1^) in rubber and rubber–*Flemingia macrophylla* plantations from April 2014 to January 2017 (mean ± SD, *n* = 3).

Soil depth (cm)	Treatments	Apr 2014	Jun 2015	Jan 2016	Aug 2016	Jan 2017
0–10 cm	R1	1.48 ± 0.03aA	1.50 ± 0.10aA	1.44 ± 0.06abA	1.50 ± 0.06aA	1.45 ± 0.04aA
	RF1	1.45 ± 0.20abA	1.45 ± 0.07aA	1.42 ± 0.15abA	1.55 ± 0.35aA	1.46 ± 0.1aA
	R2	1.48 ± 0.04aA	1.45 ± 0.07aA	1.52 ± 0.06aA	1.46 ± 0.11aA	1.55 ± 0.05aA
	RF2	1.48 ± 0.11aA	1.43 ± 0.11aA	1.50 ± 0.04aA	1.41 ± 0.13abA	1.47 ± 0.14aA
10–30 cm	R1	1.15 ± 0.10cA	1.12 ± 0.03bA	1.17 ± 0.07cA	1.16 ± 0.10bcA	1.17 ± 0.06bA
	RF1	1.15 ± 0.07cA	1.18 ± 0.09bA	1.19 ± 0.12cA	1.12 ± 0.16cA	1.10 ± 0.06bA
	R2	1.28 ± 0.07cA	1.21 ± 0.03bAB	1.29 ± 0.05bcA	1.17 ± 0.04bcB	1.22 ± 0.04bAB
	RF2	1.29 ± 0.08bcA	1.24 ± 0.06bA	1.23 ± 0.09cA	1.18 ± 0.12bcA	1.23 ± 0.09bA

R1: rubber plantations established in 2006; R2: rubber plantations established in 1994; RF1: *Flemingia macrophylla* introduced to R1 in 2010; RF2: *Flemingia macrophylla* introduced to R2 in 2010. Values within a column followed by the same letter (lower case) or within the same row (upper case) do not differ significantly at *P* ≤ 0.05.

**Table 6:** Light fraction organic nitrogen (LFON) (g kg^−1^) in rubber and rubber–*Flemingia macrophylla* plantations from June 2015 to January 2017 (mean ± SD, *n* = 3).

Soil depth (cm)	Treatments	Jun 2015	Jan 2016	Aug 2016	Jan 2017
0–10 cm	R1	0.057 ± 0.010b	0.051 ± 0.010b	0.049 ± 0.012c	0.044 ± 0.003b
	RF1	0.062 ± 0.009b	0.070 ± 0.014a	0.117 ± 0.016a	0.068 ± 0.005a
	R2	0.077 ± 0.013a	0.085 ± 0.014a	0.077 ± 0.009b	0.068 ± 0.013a
	RF2	0.078 ± 0.005a	0.069 ± 0.003a	0.077 ± 0.005b	0.051 ± 0.004b
10–30 cm	R1	0.016 ± 0.003c	0.011 ± 0.002c	0.020 ± 0.004d	0.020 ± 0.003c
	RF1	0.021 ± 0.005c	0.019 ± 0.002c	0.026 ± 0.004d	0.019 ± 0.005c
	R2	0.024 ± 0.004c	0.023 ± 0.006c	0.024 ± 0.004d	0.022 ± 0.007c
	RF2	0.025 ± 0.004c	0.021 ± 0.003c	0.027 ± 0.002d	0.020 ± 0.005c

R1: rubber plantations established in 2006; R2: rubber plantations established in 1994; RF1: *Flemingia macrophylla* introduced to R1 in 2010; RF2: *Flemingia macrophylla* introduced to R2 in 2010. Values within a column followed by the same letter do not differ significantly at *P* ≤ 0.05.

**Figure 4:**
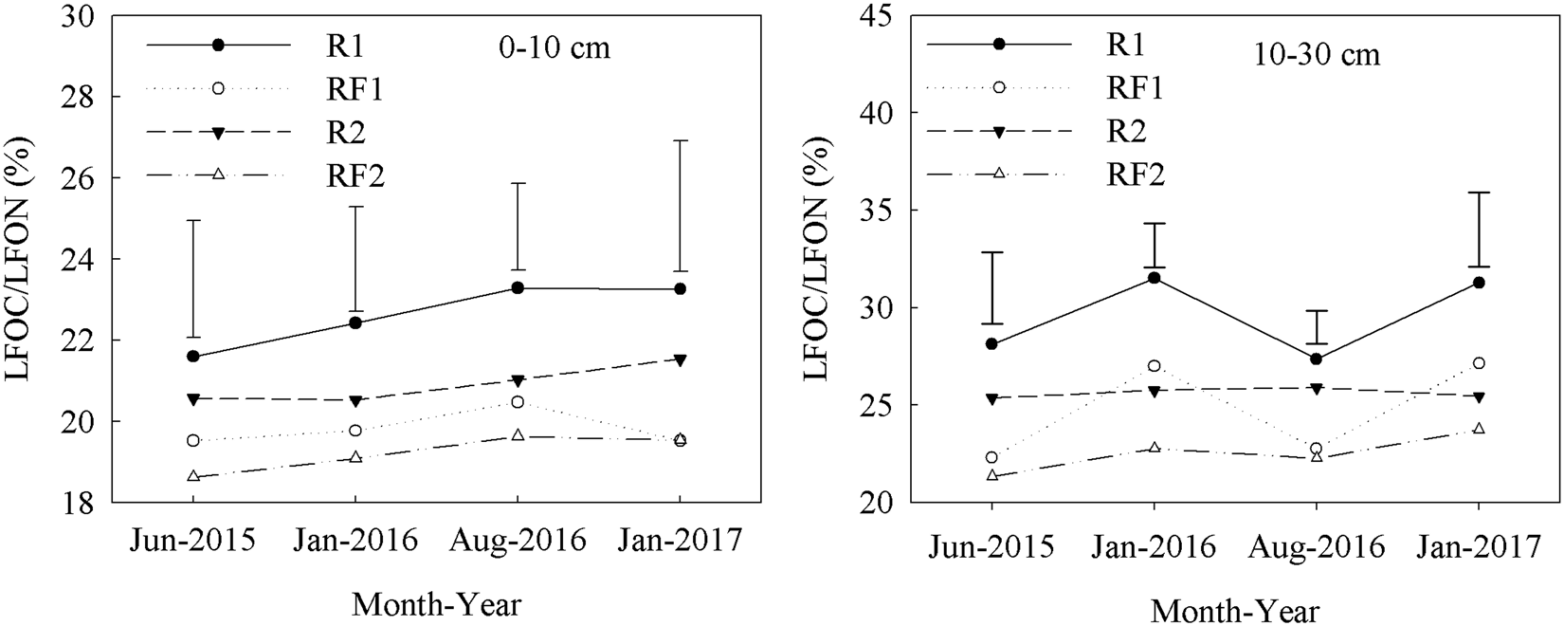
Ratio of organic C to total N in the soil light fractions (LFOC/LFON) in the rubber and rubber–*Flemingia macrophylla* plantations from June 2015 to January 2017. R1: rubber plantations established in 2006; R2: rubber plantations established in 1994; RF1: *Flemingia macrophylla* introduced to R1 in 2010; RF2: *Flemingia macrophylla* introduced to R2 in 2010. Vertical bars are LSD at *P* ≤ 0.05.

The introduction of *Flemingia macrophylla* to the rubber plantations increased MBN content and the ratios of MBN/TN in the 0–10 and 10–30 cm soil layers (Table 7 and Fig. 1). In the 0–10 cm soil layer, RF1 had consistently higher ratios of LFON/TN than R1 from January 2016 to January 2017, but no significant differences were observed between the RF2 and R2 for the duration of the study (Fig. 2). In the 10–30 cm soil layer, the introduction of *Flemingia macrophylla* to the rubber plantations had no significant effect on the ratios of LFON/TN. The introduction of *Flemingia macrophylla* to the rubber plantations decreased the ratios of LFOC/LFON in the 0–10 and 10–30 cm soil layers (Fig. 4).

**Table 7:** Soil microbial biomass nitrogen (MBN) (mg kg^−1^) in rubber and rubber–*Flemingia macrophylla* plantations from June 2015 to January 2017 (mean ± SD, *n* = 3).

Soil depth (cm)	Treatments	Jun 2015	Jan 2016	Aug 2016	Jan 2017
0–10 cm	R1	18.2 ± 1.3d	35.2 ± 14.1b	21.2 ± 3.2c	20.5 ± 4.6cd
	RF1	28.8 ± 4.6bc	42.5 ± 11.9ab	44.1 ± 10.5b	32.5 ± 5.4ab
	R2	24.0 ± 4.8c	54.7 ± 10.2a	38.6 ± 3.9b	34.0 ± 1.4ab
	RF2	34.0 ± 3.2b	55.2 ± 3.3a	59.3 ± 7.1a	36.1 ± 7.1a
10–30 cm	R1	5.3 ± 0.2e	32.7 ± 3.4b	17.1 ± 4.1c	17.0 ± 6.2de
	RF1	10.8 ± 3.3e	36.6 ± 3.9b	25.2 ± 1.7c	12.3 ± 3.9e
	R2	30.4 ± 2.1b	32.8 ± 5.9b	49.4 ± 15.5ab	27.1 ± 0.6bc
	RF2	37.1 ± 3.3a	35.8 ± 6.4b	45.8 ± 4.7b	32.2 ± 0.6ab

R1: rubber plantations established in 2006; R2: rubber plantations established in 1994; RF1: *Flemingia macrophylla* introduced to R1 in 2010; RF2: *Flemingia macrophylla* introduced to R2 in 2010. Values within a column followed by the same letter do not differ significantly at *P* ≤ 0.05.

### Relationships between soil carbon and nitrogen fractions

MBC and MBN had significant positive correlations with LFOC, LFON, and the ratios of MBC/SOC, LFOC/SOC, MBN/TN and LFON/TN (Table 8). MBC and MBN also had negative correlations with the ratio of LFOC/LFON. AN and NN had significant positive correlations with LFOC and LFON, and the ratios of LFOC/SOC and LFON/TN. Furthermore, AN and NN had significant negative correlations with LFOC/LFON.

**Table 8:** Correlation coefficients (R) among different soil carbon and nitrogen fractions in rubber and rubber–*Flemingia macrophylla* plantations from June 2015 to January 2017.

Measurement	LFON/TN	LFOC/SOC	LFOC/LFON	MBN/TN	MBC/SOC	NN	AN	LFON	MBN	LFOC	MBC
MBC	0.45*	0.40*	−0.47**	0.91***	0.95***	0.26	0.39*	0.43*	0.92***	0.39*	
LFOC	0.97***	0.99***	0.69***	0.20	0.13	0.39*	0.41*	0.99***	0.44*		
MBN	0.48**	0.44*	−0.46**	0.96***	0.85***	0.28	0.20	0.48**			
LFON	0.99***	0.98***	−0.75***	0.23	0.17	0.45**	0.41*				
AN	0.43*	0.42*	−0.45**	0.13	0.30	0.25					
NN	0.47***	0.42*	−0.55**	0.17	0.16						
MBC/SOC	0.19	0.15	0.28	0.92***							
MBN/TN	0.25	0.21	0.28								
LFOC/LFON	0.76***	0.67***									
LFOC/SOC	0.98***										
LFON/TN											

MBC: soil microbial biomass carbon; LFOC: light fraction organic carbon; MBN: soil microbial biomass nitrogen; LFON: light fraction organic nitrogen; AN: soil ammonium nitrogen; NN: soil nitrate-nitrogen; MBC/SOC: ratio of MBC to SOC; MNB/TN: ratio of MBN to TN; LFOC/LFON: ratio of LFOC to LFON; LFOC/SOC: ratio of LFOC to SOC; LFON/TN: ratio of LFON to TN. Statistical significance: *P ≤ 0.05, **P ≤ 0.01, ***P ≤ 0.001.

## Discussion

### Soil carbon and nitrogen fractions in the rubber and rubber–*Flemingia macrophylla* plantation systems

**Figure 5:**
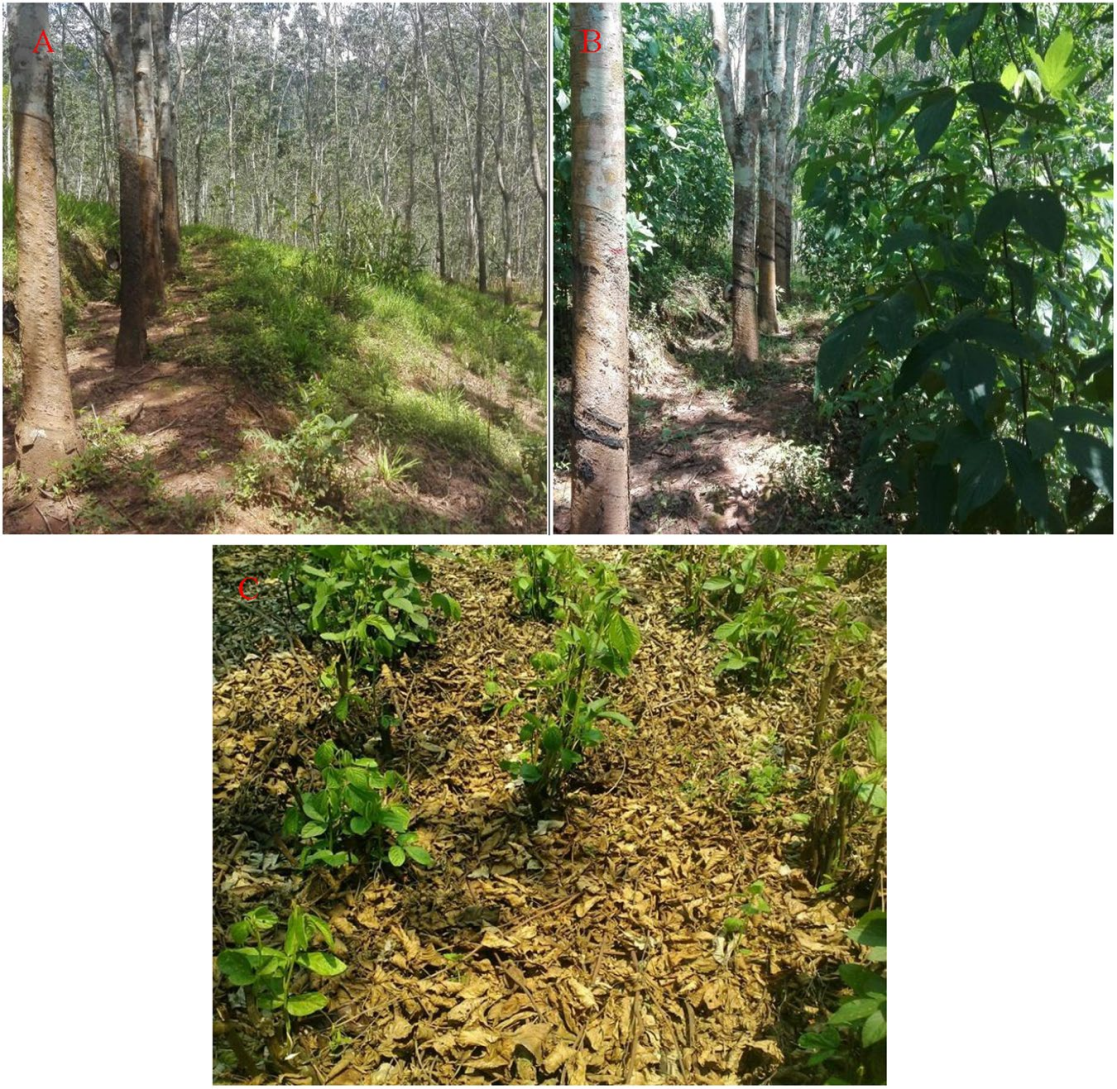
Photographs of the rubber and rubber–*Flemingia macrophylla* plantations. (**A**) rubber plantation; (**B**) rubber–*Flemingia macrophylla* plantation; (**C**) *Flemingia macrophylla* after mowing in the rubber–*Flemingia macrophylla* plantation.

Plantations with suitable native, broad-leaved species (for example, *Alnus subcordata* C. A. Mey.) along with planned forestation management could potentially rehabilitate the degraded natural forests of northern Iran^31^. Mo and Sha^32^ reported that adding carbon-sink plants into rubber plantations increased the soil organic carbon storage. The presence of legumes in semi-arid grasslands increased soil C and N storage by increasing the above- and below-ground biomass, litter biomass, plant species richness, and diversity^13^. In 2014 in present study, biomass of *Flemingia macrophylla* accumulated in the young and mature rubber plantations at 23.02 and 0.55 t ha^−1^, respectively^14^ but had no significant effect on SOC or TN contents. There are four possible explanations for this response: (1) the rubber–*Flemingia macrophylla* system had only been established for seven years, and it is unlikely that soil C and N storage would have changed significantly in such a short time; (2) the high-temperature and high-humidity environments in the rainy season would promote the decomposition of plant residues and nutrient leaching, which is not conducive to C and N storage; (3) the introduction of *Flemingia macrophylla* to the rubber plantations reduced plant species richness and diversity (Fig. 5) and the input of litter biomass of other species; (4) most rubber plantations in this area have been planted on sloping land^20^. In our study, the rubber and rubber–*Flemingia macrophylla* plantations were planted on sloping land between 47 and 58%, and the nutrients of the litter decomposition and some un-decomposed litter of *Flemingia macrophylla* would have been lost due to runoff.

While the introduction of *Flemingia macrophylla* to the rubber plantations had no significant effect on the C and N contents in the soil, the fractions of C and N changed significantly. The introduction of *Flemingia macrophylla* in the young rubber plantations was propitious for improving the labile organic C (WSOC and LFOC) and LFON contentin the 0–10 cm soil layersdue to the input of large amounts of litter from *Flemingia macrophylla*each year (Fig. 5C). The light fraction of the soil mainly consisted of plant residues, small animals, and microorganisms in various stages of decomposition. It served as a readily decomposable substrate for soil microorganisms and a short-term reservoir for plant nutrients. It potentially served as an early indicator of the effects of the management practices^33,34^. The WSOC also played a dominant role in several soil processes and was more sensitive for land use than the total SOC pool^28,35^. The WSOC indicated that the introduction of *Flemingia macrophylla* to the young rubber plantations could change the soil C fractions in the 0–10 cm soil layer within a short time. However, the introduction of *Flemingia macrophylla* into the mature rubber plantations had no significant effect on WSOC, LFOC, or LFON content in the short term, due to the lower input levels of *Flemingia macrophylla* litter each year.

In this study, the introduction of *Flemingia macrophylla* to the rubber plantations decreased AN content and increased NN content, which may have been due to the increased rate of nitrification from improved microbial activity in the rubber–*Flemingia macrophylla* systems. The NN content in soil is important for the growth of rubber trees, as well as rubber production^36^. In this region, soil NN content in rubber plantations is generally <1 mg kg^−1 ^^37^. In our study, soil NN content in the rubber plantations ranged from 0.02–0.93 mg kg^−1^. These findings suggest that *Flemingia macrophylla* is important for improving NN content in the soil of the rubber plantations^37^.

### Relationships between soil carbon and nitrogen fractions

The microbial biomass of soil controls organic matter sequestration and decomposition and is generally used as an ecological sensitivity indicator for changes in soil nutrients due to land use practices^38,39^. For example, high MBC/SOC and MBN/TN ratios indicate that the organic matter decomposed quickly^40^. In the current study, the introduction of *Flemingia macrophylla* to the rubber plantations increased MBC and MBN, along with the MBC/SOC and MBN/TN ratios in the 0–10 and 10–30 cm soil layers. These results agree with those of Wen *et al*.^41^, who suggested that the conversions of monocultures into mixed forests had a strong positive effect on soil microbial biomass, and increased the efficiency of microbes in soil carbon decomposition along the profile by improving the ratios of the MBC/SOC and MBN/TN. Interestingly, the introduction of *Flemingia macrophylla* to the rubber plantations in our study decreased the ratio of LFOC/LFON. It was observed that large amounts of leaves from the *Flemingia macrophylla* were incorporated into the soil each year, which tended to decrease the LFOC/LFON ratio in the soil due to the low C/N ratio in the leaves of *Flemingia macrophylla*. Generally speaking, the C/N ratio of *Flemingia macrophylla* is approximately 15:1^14^. Chen^42^ and Huang^43^ reported that low C/N ratios in soil could increase microbial biomass, and accelerate the decomposition of soil organic matter. We also observed a significant negative correlation between the ratio of LFOC/LFON, and MBC and MBN (*P* ≤ 0.01). These results suggest that the decreased ratios of LFOC/LFON in the rubber–*Flemingia macrophylla* plantations enhanced microbial activity.

## Conclusions

While the introduction of *Flemingia macrophylla* to the rubber plantations did not significantly increase total soil carbon or nitrogen levels over a short period, it changed the carbon and nitrogen fractions, improved labile organic carbon and nitrogen contents, and ameliorated the soil environment. We recommend that local governments and farmers in southeastern Asia use *Flemingia macrophylla* as alternative interplanted tree species within rubber plantations.

## Materials and Methods

### Description of the study site

This study was conducted in the Xishuangbanna region (21°33′N, 101°28′E; 880 to 900 m asl) of Yunnan Province in southwestern China. This region has a typical tropical monsoon climate, with an annual mean temperature of 21.8 °C. The area receives mean annual precipitation of ~1,500 mm, 80% of which occurs in the rainy season (May to October)^44^. Furthermore, Xishuangbanna contains the largest area of tropical rainforests in China. Its biodiversity is rich as it is part of the Indo-Burma world biodiversity hotspot^45^. The soil has been classified as laterite (Oxisol), which developed from arenaceous shale sediment^44,46^.

In 1991 and 2003, the tropical forests with slopes ranging from 47–58% were deforested. Sugarcane (*Saccharum officinarum* L.) was then planted annually. Rubber trees were plantedon these sites in May 1994 and 2006 at a density of 450 rubber trees ha^−1^, with 8 m spacing between adjacent rows. In accordance with the local practices for rubber trees less than three years of age, fertilizers were applied between the rubber trees at depths of 20 cm using spades at rates of 27.0 kg ha^−1^ N, 5.9 kg ha^−1^ P, and 11.2 kg ha^−1^ K, which were split into two applications per year (May and October). Once the rubber trees were more than three years of age, the fertilizer application rates changed to 54.0 kg ha^−1^ N, 11.8 kg ha^−1^ P, and 22.4 kg ha^−1^ K. The rubber plantation farmers generally sprayed sulfur powder at 30–60 kg ha^−1^ yr^−1^ to control powdery mildew on the rubber trees. Weeds in the plantations were cut using a sickle twice per year (April/May and November/December) and left on the ground. In July 2010, *Flemingia macrophylla* was introduced into the differently aged rubber plantations (4 and 16 years of age) at a density of 10,830 plants ha^−1^. From 2011 onwards, the *Flemingia macrophylla* in the different rubber plantations was cut using a sickle in December each year and left as ground cover. From 2012 onwards, given the strong biological nitrogen fixation of *Flemingia macrophylla*, no additional N was applied in the rubber–*Flemingia macrophylla* plantations. The inputs of P, K, and S in the rubber–*Flemingia macrophylla* plantations remained the same as those in the adjacent rubber plantations.

### Experimental design, sampling, and measurements

In this study, three replicate sites were selected within each rubber and rubber–*Flemingia macrophylla* plantation (Fig. 5). Each replication site consisted of 20 × 25 m^2^ survey plots (four rows of rubber trees, and three 8-m wide hedgerows) containing nine sampling subplots (8 × 6 m^2^), with three located at each slope position (upper, middle, and lower slope).

For each of the nine subplots, soil samples were collected using a soil auger, avoiding the fertilization holes, at two depths (0–10 cm and 10–30 cm) after carefully removing the litter-fall and/or grass layer. For each replicate site in the different rubber and rubber–*Flemingia macrophylla* plantations, soil core samples were collected in April 2014, June 2015, January and August 2016, and January 2017. The nine soil cores were combined into a composite sample, which were air-dried, ground, and sieved (at <2 mm) for analysis of ammonium nitrogen (AN) and nitrate-nitrogen (NN), and the light fraction organic carbon (LFOC) and nitrogen (LFON). The sieved samples were sieved again (at <0.25 mm) for determination of soil organic carbon (SOC) and total nitrogen (TN). For soil water-soluble organic carbon (WSOC), microbial biomass carbon (MBC) and nitrogen (MBN) measurements, the samples were taken to the laboratory and stored at 4 °C for subsequent analyses.

The SOC and TN of the bulk soil were determined using a Vario MAX CN-Analyzer (Elementar Analysensysteme GmbH, Germany). A density fractionation scheme for light was used following the method described by Gregorich and Ellert^47^. During fractionation, 25 g of air-dried soil was shaken with 50 mL of NaI solution (sp.Gr. = 1.70) for 60 min. After centrifugation, the supernatant was passed through a Millipore filter (0.45 μm) and the light fraction collected. The soil residue in the centrifuge was extracted again with NaI, and the additional light fraction collected. The light fraction was oven-dried at 60 °C of 72 h. The concentration of organic carbon and nitrogen was determined by dry combustion using a Vario MAX CN-Analyzer (Elementar Analysensysteme GmbH, Germany). Dried samples, each weighing 5 g, were added to 50 mL of 2 M KCl, shaken for one hour, and analyzed with an Auto Analyzer 3 (SEAL Analytical GmbH, Germany) to determine AN and NN contents^34,48^. Microbial biomass carbon (MBC) and nitrogen (MBN) in the soil were estimated using a fumigation-extractionmethod^49^ that included a purified CHCl_3_ treatment, followed by a 0.5 M K_2_SO_4_ extraction of fumigated and unfumigated soil^50^. After which, soil samples (equivalent to 25 g of dry soil weight) were fumigated for 24 h at 25 °C with CHCl_3_ (ethanol-free). Following fumigant removal, the soil was extracted with 100 mL of 0.5 M K_2_SO_4_ by shaking for 1 h at 200 rpm, followed by filtering. The non-fumigated portions were extractedat similar time intervals. Following the extraction, MBC and MBN contents were measured by determining the C and N masses in the filtrate using a Vario TOC cube-Analyzer (Elementar Analysensysteme GmbH, Germany). Microbial biomass carbon was calculated as follows: MBC = (Corg(fum) − Corg(non))/0.38^51^. Microbial biomass nitrogen wascalculated as follows: MBN = (TN(fum) − TN(non))/0.45^51^. The ratios of MBC to total carbon (MBC/SOC) and MBN to total nitrogen (MBN/TN) were then calculated. Water-soluble organic carbon (WSOC) was extracted from field-moist samples within 24 h of sampling by shaking 15 g soil with 30 mL distilled water for 2 h at 25 °C, followed by centrifugation at 5000 r min^*−*1^ at 4 °C for 15 min. The supernatant was filtered through a 0.45 μm carbon-free membrane. The filtrates were stored at 4 °C and analyzed within 24 h using a Vario TOC cube-Analyzer (Elementar Analysensysteme GmbH, Germany)^35^.

### Statistical analysis

The data were subjected to analysis of variance (ANOVA) using SAS statistical analysis software version 8.0. One-factor ANOVA was deployed to compare treatment effects. The least significant difference (LSD at 0.05 level of probability) test was applied to assess the differences between means. Pearson’s coefficient analysis was used for correlation.
